# Ambient Particulate Matter (PM_2.5_/PM_10_) Exposure and Emergency Department Visits for Acute Myocardial Infarction in Chaoyang District, Beijing, China During 2014: A Case-Crossover Study

**DOI:** 10.2188/jea.JE20150209

**Published:** 2016-10-05

**Authors:** Qian Zhang, Weipeng Qi, Wei Yao, Mei Wang, Yiyong Chen, Yujie Zhou

**Affiliations:** 1 Department of Emergency, Anzhen Hospital, Capital Medical University, Beijing, China; 2 Department of Occupational and Environmental Health Science, School of Public Health, Peking University, Beijing, China

**Keywords:** PM, AMI, STEMI, NSTEMI, time-stratified case-crossover study

## Abstract

**Background:**

Epidemiology studies have shown a consistently increased risk of acute myocardial infarction (AMI) correlated with particulate matter (PM) exposure. However, little is known about the association with specific AMI subtypes. In this work, we investigated the association between short-term PM exposure and emergency department visits (EDVs) for AMI, ST-elevation myocardial infarction (STEMI), and non-ST-elevation myocardial infarction (NSTEMI).

**Methods:**

We based this case-crossover study on 2749 patients from Chaoyang District hospitalized with AMI in Anzhen Hospital during 2014. Meteorological and air pollution data were collected during this period. We used a time-stratified case-crossover design with lag model, adjusted for meteorological conditions and/or other gaseous pollutants, to estimate risk of EDVs for AMI, STEMI, and NSTEMI. We conducted stratified analyses by gender, age, season, and comorbid conditions to examine potential effect modification.

**Results:**

We found that each 10 µg/m^3^ increment of PM_2.5_ concentration (1-day lagged) was associated with an increased risk of EDVs for STEMI (OR 1.05; 95% CI, 1.00–1.11). We found no association of PM_2.5_ concentration with overall AMI or NSTEMI. No effect modification was found when stratified by gender, season, or comorbid conditions, even though the effect size was larger in patients who were male, smokers, and comorbid with hypertension. Patients aged ≥65 years showed a significantly increased risk of STEMI associated with PM_2.5_ in the previous day than those aged <65 years.

**Conclusions:**

Our study indicated a transient effect of short-term PM_2.5_ exposure on EDVs for STEMI. Patients aged ≥65 years appeared to be particularly susceptible. Our findings suggest that studies of the association between PM exposure and AMI should consider AMI subtypes, lag times, and individual characteristics.

## INTRODUCTION

Ambient particulate matter (PM) air pollution has become one of the most important issues in Beijing over the last few years. For example, Guo et al^1^ reported that interquartile range (IQR) increases in PM_2.5_, PM_10_, SO_2_, and NO_2_ were related to years of life lost (YLL) increases of 15.8, 15.8, 16.2, and 15.1 years, respectively, in Beijing during 2004–2008. Generally, the ambient PM pollutants are defined according to their aerodynamic diameter: PM of aerodynamic diameter ≤10 µm was defined as PM_10_ (also called inhalable particles), and PM ≤2.5 µm was defined as PM_2.5_ (fine particles). In 2013, annual mean concentrations of PM_2.5_ and PM_10_ in Beijing were 89.5 µg/m^3^ and 108.1 µg/m^3^, respectively, which were still far above the level recommended by the WHO’s guidelines on air quality (10 µg/m^3^ for PM_2.5_ and 20 µg/m^3^ for PM_10_).

In 2004, the AHA Scientific Statement^2^ concluded that exposure to PM air pollution contributes to cardiovascular morbidity and mortality, especially for myocardial infarction (MI), stroke, cardiac arrhythmia, and heart failure. In the Harvard Six Cities Study^3^ and the American Cancer Society Study on Particulate Air Pollution and Mortality,^4^ PM_2.5_ was substantially associated with cardiovascular mortality. However, their findings were based on long-term air pollution exposure. A substantial number of case-crossover studies have explored the association between short-term ambient PM exposure and risk of acute myocardial infarction (AMI).^5^^–^^16^ The majority of these studies indicated that elevated concentrations of particulate matter in the air may transiently elevate the risk of MIs with short-term exposure, although odds ratios (ORs) are relatively small.

AMIs occur when a coronary artery is suddenly partially or completely blocked by thrombus, causing the related heart muscle to become infarcted. It is generally classified into STEMI and NSTEMI according to electrocardiogram manifestations. STEMI shows ST segment elevation in ECG and later progress to a Q-wave, which is not found in NSTEMI. Mechanistically, STEMI differ from NSTEMI in that STEMI develops a complete major coronary arterial occlusion following plaque rupture, causing full-thickness damage to the heart muscle rather than the subtotal coronary occlusion and partial damage seen with NSTEMI.^17^ Therefore, the PM-associated risk estimate differences for STEMI and NSTEMI may elucidate pathophysiological mechanisms involved in how PM triggers MI.

The issue of sensitivity to ambient PM air pollution is often in the research spotlight. Effect modification of the association between PM and AMI by gender, age, season, smoking history, and comorbid conditions, such as hypertension and diabetes, have been reported in previous studies^6^^,^^9^^,^^11^^,^^12^^,^^15^; however, findings have been inconsistent. Hence, there is a need to consider these individual characteristics in our study.

We used a time-stratified case-crossover design with lag model to analyze the relationship between ambient particulate air pollution and emergency department visits (EDVs) for AMI adjusted for meteorology data and/or other pollutants, with a specific focus on highlighting the differences in risk estimates between STEMI and NSTEMI. Further, we explored the associations stratified by several individual characteristics.

## MATERIALS AND METHODS

### Study population and outcome definition

**Figure 1.**
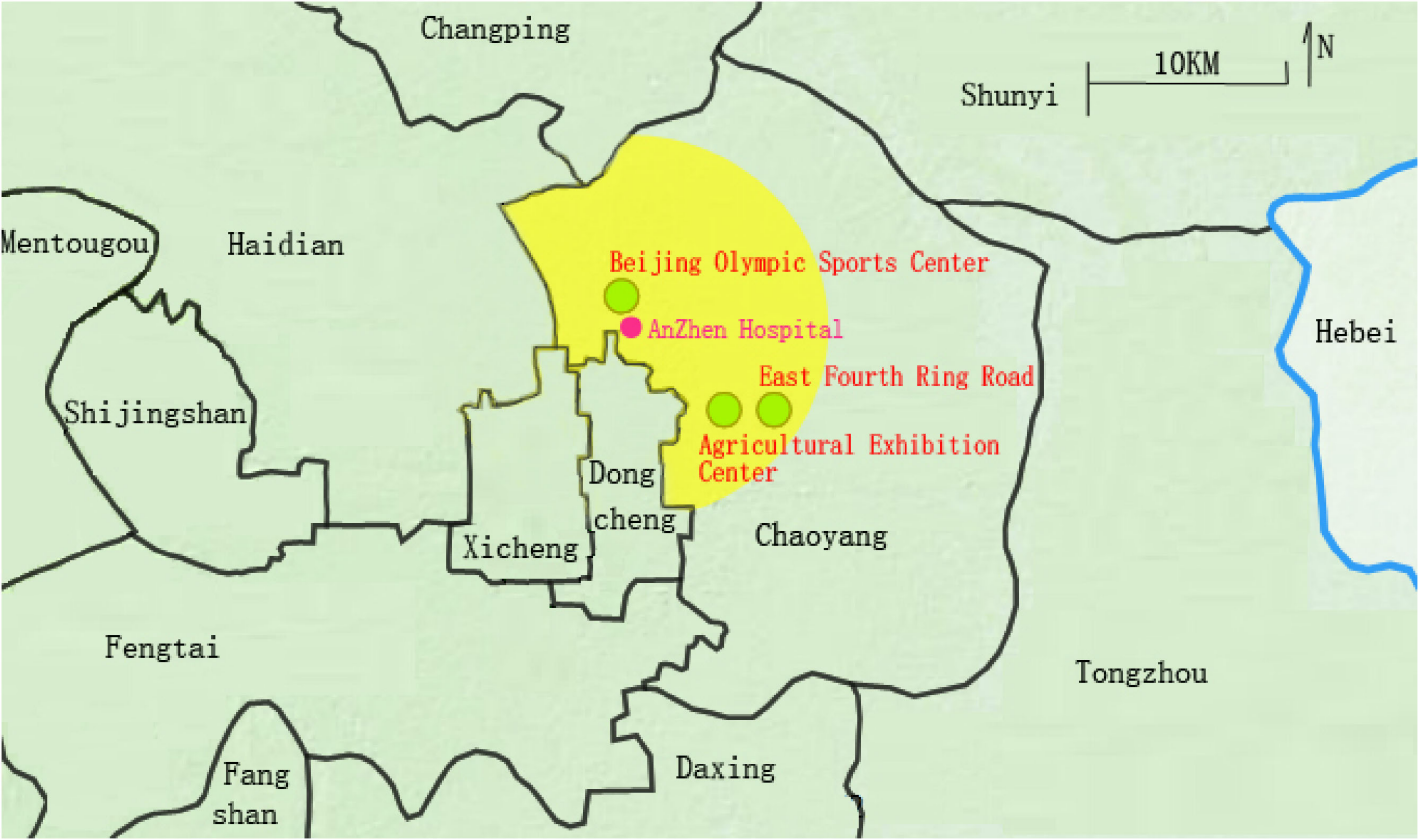
Map of study area showing PM monitoring sites.

Data on EDVs for AMI were gathered from residents of northern Chaoyang District who were hospitalized with AMI at Beijing Anzhen Hospital during 2014. Beijing Anzhen Hospital is the AMI-designated hospital for local residents in our study area (shown in Figure 1). All included cases lived within 10 km of our hospital. The information obtained for each case were name, age, gender, residence address, comorbid conditions, and clinical events. We excluded patients who were not currently living in this region and who had experienced other AMI events within 28 days prior. The data set included clinical events, pollutants, meteorological conditions, and individual characteristics, which were linked through admission date. We categorized admissions according to the primary diagnosis International Classification of Diseases, Tenth Revision, Clinical Modification (ICD-10-CM) codes for AMI (I21.0–21.3), STEMI (I21.4), and NSTEMI (I20.0/20.1/20.9). This study was approved by the Beijing Anzhen Hospital Research Subjects Review Board.

### Air pollution exposure and weather condition assessment

Information on daily mean temperature and relative humidity was obtained from the China Meteorological Center from January 1 to December 31, 2014. We collected ambient pollutant measurements during the study period from three monitoring sites of the Beijing Municipal Environmental Monitoring Center—Beijing Olympic Sports Center, Agricultural Exhibition Center, and East Fourth Ring Road, which were uniformly distributed in our study area. We used mean daily concentrations of PM_2.5_, PM_10_, nitrogen dioxide (NO_2_), sulfur dioxide (SO_2_), carbon monoxide (CO), and ozone (O_3_) in our subsequent analyses.

### Study design

We used a time-stratified case-crossover design, which had previously been used in various studies^7^^,^^8^^,^^11^^,^^15^^,^^16^^,^^18^ of ambient PM exposure and myocardial infarction. In this design, PM concentrations were compared during the period of patients experiencing AMI (the case period) with the times not experiencing AMI (the control period).^19^ We estimated the relative risk of AMI comparing PM exposure during case periods and control periods. Control periods were matched to case periods using the time-stratified method, with 28 days strata by same day of a week and calendar month (eg, the first stratum was January 1 to January 28, 2014). Each case day had 3 matching control days, with irregular occurrences prior or after the case day. By matching on the day of the week, we controlled confounding due to fluctuant pollutants levels on different week days. The design can also control confounders related to individual characteristics, since each case serves as its own control, as well as secular trends and seasonal patterns.^20^ Weather conditions, such as temperature and relative humidity, were time-dependent variables. We adjusted for temperature and relative humidity using a natural smooth spline with 3 degrees of freedom in each model.

### Statistical analysis

Conditional logistic regression models were used to estimate the association between pollutants and AMI outcomes. Spearman correlation coefficients were used to summarize the correlations between weather conditions and air pollutants. We presented ORs and their 95% confidence intervals (CIs) scaled to each 10 µg/m^3^ change of PM concentrations observed during our study period. We investigated the risk of AMI, STEMI, and NSTEMI associated with each 10 µg/m^3^ increase in mean daily PM concentration at 0–5 separated lagged days to get the greatest risk estimates. To assess the stability of our PM relative risk estimates after adjusting for gaseous pollutant concentrations, we used single and multiple pollutant models to determine the independent/combined effects of air pollutants on AMI outcomes. Stratification was done by gender, age, season (cold season: October–March; warm season: April–September), smoking history, and comorbidities (diabetes and hypertension) to better elucidate the effect modifications to PM-AMI associations. We added interaction term for the PM concentrations and personal characteristics (eg, age × PM_2.5_) to the same conditional logistic regression model described above.

All analyses were conducted using SPSS (version 19.0; IBM Corp, Armonk, NY, USA). *P*-values <0.05 were considered statistically significant.

## RESULTS

**Figure 2.**
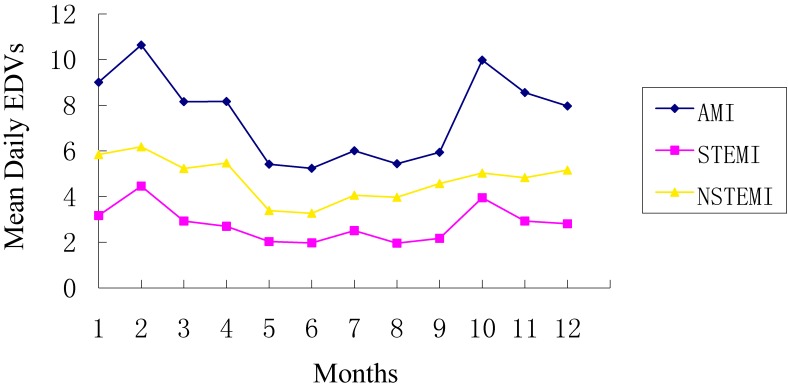
Time series of mean daily EDVs for AMI, STEMI and NSTEMI during 2014. AMI, acute myocardial infarction; EDV: emergency department visit; NSTEMI, non-ST-elevation myocardial infarction; STEMI, ST-elevation myocardial infarction.

During the study period, a total of 2749 patients from our study area who were admitted to Beijing Anzhen Hospital diagnosed with AMI (1016 STEMI patients and 1733 NSTEMI patients) were included in our analysis. As is shown in Table 1, about 58.64%, 62.30%, and 56.49% of the cases were male and 54.60%, 48.52%, and 58.17% of the cases were aged ≥65 years for AMI, STEMI, NSTEMI, respectively. The proportions of patients with a history of smoking, hypertension, and diabetes were 56.86%, 60.86%, and 30.48%, respectively. Figure 2 showed the time series descriptive statistics of EDVs for AMI, STEMI, and NSTEMI from January 1 through December 31, 2014.

**Table 1.** General characteristics of the study population

	AMI *n* = 2749	STEMI *n* = 1016	NSTEMI *n* = 1733
Gender			
Male	1612 (58.64%)	633 (62.30%)	979 (56.49%)
Female	1137 (41.36%)	383 (37.70%)	754 (43.51%)
Age, years			
<65	1248 (45.40%)	523 (51.48%)	725 (41.83%)
≥65	1501 (54.60%)	493 (48.52%)	1008 (58.17%)
Season			
Warm	1227 (44.63%)	421 (41.44%)	806 (46.51%)
Cold	1522 (55.37%)	595 (58.56%)	927 (53.49%)
Smoking			
Yes	1563 (56.86%)	611 (60.13%)	952 (54.93%)
No	1186 (43.14%)	405 (39.86%)	781 (45.07%)
Hypertension			
Yes	1673 (60.86%)	541 (53.24%)	1132 (65.32%)
No	1076 (39.14%)	475 (46.75%)	601 (34.68%)
Diabetes			
Yes	838 (30.48%)	329 (32.38%)	509 (29.37%)
No	1911 (69.52%)	687 (67.62%)	1224 (70.63%)

AMI, acute myocardial infarction; NSTEMI, non-ST-elevation myocardial infarction; STEMI, ST-elevation myocardial infarction.

The distributions of ambient PM concentrations, gaseous pollutants concentrations, and meteorological conditions during the study period (January to December 2014) are shown in Table 2. The daily mean concentrations for PM_2.5_, PM_10_, SO_2_, NO_2_, CO, and O_3_ were 84.54, 116.38, 20.56, 54.69, 1266.70, and 56.24 µg/m^3^, respectively. The mean daily PM_2.5_ concentration showed a large variation, ranging from 5.2 to 392.6 µg/m^3^, and so did PM_10_. The mean daily PM_2.5_ concentration far exceeded the World Health Organization (WHO) recommendations, with only 63 days (17.3%) falling below the WHO recommended value.

**Table 2.** Descriptive statistics of air pollutants and weather conditions

	P_25_	P_50_	P_75_	Minimum	Maximum	Mean (SD)
Air pollutant concentrations, µg/m^3^						
PM_2.5_	32.25	66.50	114.55	5.20	392.60	84.54 (69.01)
PM_10_	74.28	116.60	182.82	8.90	448.60	116.38 (74.67)
SO_2_	5.20	11.00	26.90	2.00	133.10	20.56 (23.33)
NO_2_	38.65	49.40	67.50	8.10	135.90	54.69 (23.79)
CO	646.30	996.20	1574.10	222.10	5229.80	1266.70 (875.26)
O_3_	13.78	28.35	47.40	2.60	188.00	56.24 (37.94)
Meteorological factors						
Temperature, °C	2.50	14.00	23.00	−7.00	31.00	13.36 (10.94)
Relative humidity, %	37.00	50.00	65.00	8.00	93.00	50.75 (19.08)

SD, standard deviation.

Table 3 presents Spearman correlation coefficients between air pollutants and meteorological conditions. PM_2.5_/PM_10_ were positively correlated with SO_2_, CO, NO_2_, and relative humidity but showed inverse correlation with O_3_ (*P* < 0.05). This is probably due to the fact that, as one of several air components of the photochemical smog, the mechanisms of ozone formation and destruction are complex and varied. This correlation indicates that we should adjust for other pollutants and meteorological confounders when studying the association between particulate air pollution and AMI.

**Table 3.** Spearman correlation coefficients between air pollutants and meteorological conditions

	PM_2.5_	PM_10_	SO_2_	CO	NO_2_	O_3_	T	RH
PM_2.5_	1.000	.883**	.488**	.858**	.678**	−.182**	−.028	.506**
PM_10_		1.000	.555**	.738**	.733**	−.164**	−.032	.283**
SO_2_			1.000	.682**	.667**	−.484**	−.615**	−.267**
CO				1.000	.766**	−.483**	−.331**	.363**
NO_2_					1.000	−.554**	−.313**	.192**
O_3_						1.000	.779**	.032
T							1.000	.390**
RH								1.000

RH, relative humidity; T, temperature.

**P < 0.01.

**Figure 3.**
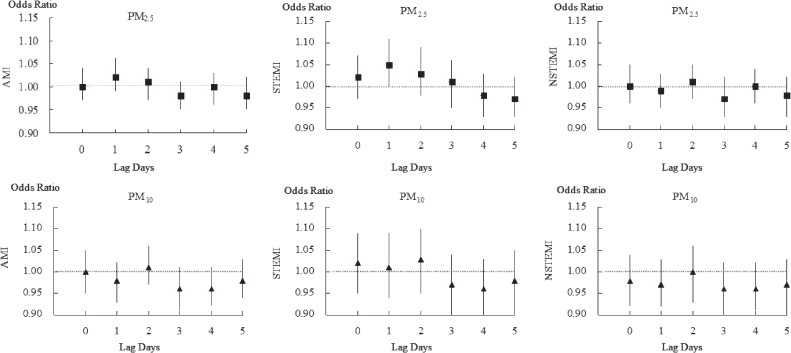
Association between each 10 µg/m^3^ increment in PM concentrations and odds ratio for emergency department visits for AMI, STEMI, and NSTEMI in 0–5 lag days. AMI, acute myocardial infarction; NSTEMI: non-ST-elevation myocardial infarction; STEMI: ST-elevation myocardial infarction. No associations were found between emergency department visits for overall AMI, NSTEMI, and any of the lagged PM_2.5_/PM_10_ concentrations. Each increment of 10 µg/m^3^ in PM_2.5_ concentration (1-day lagged) was associated with a significantly increased risk of STEMI, which indicated a transient effect of short-term PM_2.5_ exposure on emergency department visits for STEMI.

We then separately estimated the risk of EDVs for AMI, STEMI, and NSTEMI in relation to each 10 µg/m^3^ increment in daily mean concentrations at lagged 0–5 days in the single pollutant model, after adjusting for meteorological conditions (Figure 3 and Table 4). We found no associations between EDVs for overall AMI, NSTEMI, and any of the lagged PM_2.5_/PM_10_ concentrations. However, we found that each increment of 10 µg/m^3^ in 1-day-lagged PM_2.5_ concentration was associated with a significantly increased risk of STEMI (OR 1.05; 95% CI, 1.00–1.11). As for PM_10_, we did not find any associations between pollution levels and EDVs for STEMI and NSTEMI.

**Table 4.** Estimated odds ratios for each 10 µg/m^3^ increase in PM_2.5_ levels in relation to AMI, STEMI, and NSTEMI for different lag days

	Lag period	AMI OR (95% CI)	STEMI OR (95% CI)	NSTEMI OR (95% CI)
PM_2.5_	0	1.00 (0.97 1.04)	1.02 (0.97 1.07)	1.00 (0.96 1.05)
	1	1.02 (0.99 1.06)	1.05 (1.00 1.11)*	0.99 (0.95 1.03)
	2	1.01 (0.97 1.04)	1.03 (0.98 1.09)	1.01 (0.97 1.05)
	3	0.98 (0.95 1.01)	1.01 (0.95 1.06)	0.97 (0.93 1.02)
	4	1.00 (0.96 1.03)	0.98 (0.93 1.03)	1.00 (0.96 1.04)
	5	0.98 (0.95 1.02)	0.97 (0.93 1.02)	0.98 (0.93 1.02)

PM_10_	0	1.00 (0.95 1.05)	1.02 (0.95 1.09)	0.98 (0.92 1.04)
	1	0.98 (0.93 1.02)	1.01 (0.94 1.09)	0.97 (0.92 1.03)
	2	1.01 (0.97 1.06)	1.03 (0.95 1.10)	1.00 (0.93 1.06)
	3	0.96 (0.91 1.01)	0.97 (0.90 1.04)	0.96 (0.90 1.02)
	4	0.96 (0.92 1.01)	0.96 (0.90 1.03)	0.96 (0.91 1.02)
	5	0.98 (0.94 1.03)	0.98 (0.91 1.05)	0.97 (0.91 1.03)

AMI, acute myocardial infarction; STEMI, ST-elevation myocardial infarction; NSTEMI, non-ST-elevation myocardial infarction.

*P < 0.05, adjusted for temperature and relative humidity.

In the multiple-pollutant models, after controlling for SO_2_, NO_2_, and CO concentrations, we still found no association between PM_2.5_ concentrations and risk for overall AMI (Table 5). In a two-pollutant model, after controlling for SO_2_, EDVs for STEMI were significantly associated with PM_2.5_ concentration (OR 1.06; 95% CI, 1.00–1.12). However, no association was found in other multiple-pollutant models.

**Table 5.** Association between each 10 µg/m^3^ increase in PM_2.5_ concentration in the previous 24 h and emergency department visits for AMI, STEMI, and NSTEMI in single- and multiple-pollutant models

	AMI		STEMI		NSTEMI	
		
	OR	95% CI	OR	95% CI	OR	95% CI
PM_2.5_	1.01	(0.96–1.05)	1.05*	(1.00–1.11)	0.98	(0.92–1.03)
PM_2.5_ + SO_2_	1.02	(0.97–1.06)	1.06*	(1.00–1.12)	0.99	(0.93–1.06)
PM_2.5_ + NO_2_	1.02	(0.97–1.06)	1.04	(0.97–1.10)	0.98	(0.92–1.03)
PM_2.5_ + CO	1.01	(0.96–1.05)	1.03	(0.97–1.10)	0.99	(0.94–1.05)
PM_2.5_ + O_3_	1.01	(0.97–1.06)	1.04	(0.99–1.12)	0.97	(0.92–1.03)
PM_2.5_ + SO_2_ + NO_2_ + CO	1.01	(0.97–1.05)	1.04	(0.99–1.12)	0.98	(0.92–1.03)

AMI, acute myocardial infarction; CI, confidence interval; NSTEMI, non-ST-elevation myocardial infarction; OR, odds ratio; STEMI, ST-elevation myocardial infarction.

*P < 0.05, adjusted for temperature and relative humidity.

We only included STEMI in our stratified analysis by several factors (gender, age, season, and comorbidities) to examine potential effect modification, since we found association with this infarction type only (shown in Table 6). Patients aged ≥65 years had a significantly greater risk of STEMI associated with each increment of 10 µg/m^3^ in PM_2.5_ concentration in the previous 1 day than in those aged <65 years (OR 1.15; 95% CI, 1.08–1.23). Although we found no effect modification by gender, season, or comorbid conditions, risk estimates associated with EDVs for STEMI were all statistically significant and slightly larger for males, those aged ≥65 years, smokers, and those with comorbid hypertension.

**Table 6.** Risk of emergency department visits for STEMI associated with each 10 µg/m^3^ increase in PM_2.5_ concentration in the previous 24 h stratified by age, sex, season, and comorbid conditions

Characteristic		OR (95% CI)	*P*-Value for interaction
Age, years	<65	0.97 (0.90–1.05)	0.04*
	≥65	1.15 (1.08–1.23)	
Sex	Male	1.08 (1.02–1.15)	0.68
	Female	1.01 (0.93–1.09)	
Season	Winter	1.06 (0.99–1.14)	0.85
	Summer	1.03 (0.96–1.10)	
Diabetes	Yes	1.02 (0.94–1.11)	0.85
	No	1.06 (1.00–1.13)	
Hypertension	Yes	1.10 (1.03–1.17)	0.49
	No	1.01 (0.93–1.08)	
Smoking	Yes	1.08 (1.01–1.15)	0.77
	No	1.03 (0.95–1.10)	

STEMI, ST-elevation myocardial infarction.

*P < 0.05, adjusted for temperature and relative humidity.

## DISCUSSION

Using data of hospital admissions for AMI in Northern Chaoyang District, Beijing, we found that short-term PM_2.5_ exposure was not associated with increased risk of overall AMI, but was transiently associated with EDVs for STEMI (1-day lagged), particularly for elderly patients (≥65 years). We found no association between PM_10_ and risk of overall AMI, STEMI, or NSTEMI.

PM air pollution is an important issue in relation to its various adverse effects on human health, particularly on the respiratory and cardiovascular systems. There are several biological pathways linking PM exposure with cardiovascular diseases,^21^ including: 1) direct translocation of PM or its organic metallic constituents into blood, causing systematic inflammation; 2) release of proinflammatory mediators or vasculoactive molecules from lung cells, resulting in systemic oxidative stress and inflammation; and 3) interactions between PM and lung receptors or nerves, disrupting systemic autonomic nervous system balance. These pathways may contribute to endothelial dysfunction, vasoconstriction, plaque vulnerability, thrombogenecity, and atherosclerosis progression, which may explain the mechanisms underlying our results.

Our crucial finding was that 10 µg/m^3^ increments in PM_2.5_ concentration were not associated with risk of overall EDVs for AMI but were associated with an increased risk of STEMI (1-day lagged). Our findings are consistent with those from a previous study by Gardner et al,^15^ which reported a significant 18% increase in the risk of STEMI associated with each 7.1 µg/m^3^ increase in PM_2.5_ concentration, while they found no association between PM_2.5_ concentration and NSTEMI. Previous studies suggested that coagulation/inflammation pathways may be acutely affected by PM exposure.^21^ Short-term exposure to elevated PM_2.5_ levels can enhance blood coagulability, thrombosis, endothelial dysfunction, and plaque vulnerability. Plaque rupture could have a greater chance to develop occlusive artery thrombus under such circumstances. A study^22^ during the 2008 Beijing Olympic Games demonstrated that short-term elevated PM concentrations were positively correlated with elevated circulating markers of platelet and endothelial cell activation. Since plaque rupture and subsequent thrombosis of previously narrowed or unstable plaque-lined arteries is a common proximal event in the evolution of STEMI and NSTEMI,^23^ exposure to high levels of PM (especially PM_2.5_) are more likely to result in complete coronary arterial occlusion with full thickness damage of myocardium and highly elevated enzyme or troponin levels in STEMI than NSTEMI.

Our findings also indicated that only PM_2.5_ exposure (1-day lagged), not PM_10_, showed an association with EDVs for STEMI. PM_2.5_ exerts greater toxicity than PM_10_ in that it can reach a larger surface area, which makes it an excellent carrier for harmful substances like metals, organic compounds, and microorganisms. Further, it is small in size, which allows it to easily penetrate deep into the human respiratory tract and transfer into systemic circulation. This is consistent with evidence from Mustafic et al,^24^ who reported that PM was associated with an increase in MI risk, with ORs of 1.006 (95% CI, 1.002–1.009) for PM_10_ and 1.025 (95% CI, 1.015–1.036) for PM_2.5_.

We only found a statistically significant association between EDVs for STEMI and PM_2.5_ levels in the previous day. Our findings suggest a transient effect of PM on AMI, which is consistent with the results of several previous studies. Gardner et al^15^ reported a significant association of PM_2.5_ and STEMI in the previous hour prior to acute coronary syndrome onset but not with exposure in other times. Rich et al^11^ noted that each 10.8 µg/m^3^ increase in PM_2.5_ concentration in the 24 hours before arriving at the emergency department for MI was associated with an increased risk of a transmural infarction. In addition, in a case-crossover analysis of the MINAP database,^16^ the immediate increase in risk of AMI was followed by reductions in risk at longer lags; there was no evidence of any net excess risk associated with the five pollutants studied over a 72-hour period, which indicated only transient associations. Peters et al^5^ also found that elevated concentrations of fine particles in the air may transiently elevate the risk of MIs within a few hours and 1 day after exposure.

Our preliminary findings suggested that patients aged ≥65 years showed a significantly increased risk of STEMI associated with PM_2.5_ in the previous 24 hours. This vulnerability of elderly people is well documented in previous studies. In a study about air pollution and myocardial infarction in Rome,^6^ the association with PM tended to be stronger among people older than 74 years of age (OR 1.046; 95% CI, 1.005–1.089) than among younger people. In a study on the effects of air pollution on hospitalizations for cardiovascular disease in Australian and New Zealand Cities,^9^ the increases in hospital admissions for AMI were greater in the elderly (≥65 years) than in the younger age group (15–64 years). The results of the RISCAT study^12^ also indicated that elderly persons were more susceptible than younger people. The elderly are a frailer population and likely to have preexisting cardiovascular problems (eg, atherosclerosis plaque), which can increase susceptibility to ambient air pollution. Under such conditions, short-term PM_2.5_ can increase plaque vulnerability, platelet activation, and blood coagulation, easily triggering AMI events immediately after plaque rupture and thrombosis formation. There was a weaker indication of a larger effect on patients who were male, smokers, and who had comorbid hypertension, but no evidence of effect modification by age, smoking, or hypertension was found after a formal test for interaction. Conflicting findings regarding these effects have been reported in previous studies.^6^^,^^11^^,^^15^^,^^25^^,^^26^

### Study limitations

Several limitations should be considered when interpreting our results. First, we used ambient PM concentration from the monitoring sites to represent the background exposure of the general population, regardless of the individual time-activity patterns and heterogeneous exposure across the area. This might have consequently given rise to non-differential exposure misclassification and led to underestimation of the true relative risk. We also took limited factors into consideration due to confined existing data of cases hospitalized in our study during 2014. Linking daily mean air pollution concentrations to AMIs also precludes accurate assessment of the time of symptom onset and the delay from symptom onset to emergency department visits. Further, our study period (1 year) might be too short to obtain persuasive findings in this study.

### Conclusions

Our study found that short-term exposure to elevated PM levels in the previous day was associated with increased EDVs for STEMI, particularly for elderly patients (≥65 years). The transient effect of air pollution on STEMI was observed for PM_2.5_ but not for PM_10_. We noted a larger effect on patients who were male, smokers, and who had comorbid hypertension, even though we found no evidence of effect modification by gender, season, or comorbid conditions. Our findings imply the necessity of emphasis on AMI subtypes, lag times, and individual characteristics for future mechanism-focused research on the association between air pollution and AMI.
